# Association of hemoglobin concentration with handgrip strength in relation to hepatocyte growth factor levels among elderly Japanese men aged 60–69 years: a cross-sectional study

**DOI:** 10.1186/s12199-018-0744-x

**Published:** 2018-11-06

**Authors:** Yuji Shimizu, Hirotomo Yamanashi, Yuko Noguchi, Jun Koyamatsu, Mako Nagayoshi, Kairi Kiyoura, Shoichi Fukui, Mami Tamai, Shin-Ya Kawashiri, Kazuhiko Arima, Takahiro Maeda

**Affiliations:** 1 Department of Community Medicine, Nagasaki University Graduate School of Biomedical Sciences, Nagasaki-shi, Sakamoto 1-12-4, Nagasaki, 852-8523 Japan; 2 Department of Cardiovascular Disease Prevention, Osaka Center for Cancer and Cardiovascular Disease Prevention, Osaka, Japan; 3 Department of General Medicine, Nagasaki University Hospital, Nagasaki, Japan; 4 Department of Island and Community Medicine, Nagasaki University Graduate School of Biomedical Sciences, Nagasaki, Japan; 5 Department of Immunology and Rheumatology, Nagasaki University Graduate School of Biomedical Sciences, Nagasaki, Japan; 6 Department of Public Health, Nagasaki University Graduate School of Biomedical Sciences, Nagasaki, Japan

**Keywords:** Hemoglobin, Handgrip, Hepatocyte growth factor

## Abstract

**Background:**

Hemoglobin concentration reportedly is positively associated with muscle strength, for example, handgrip strength. However, hemoglobin cannot repair muscle directly, but is beneficial only in a supportive role. Since hepatocyte growth factor (HGF) regulates muscle satellite cell production and differentiation, which is stimulated by organ injury, the supportive effect of hemoglobin should thus be stronger for participants with high HGF than for those with low HGF. However, the association between hemoglobin concentration and handgrip strength in relation to HGF levels remains unknown.

**Methods:**

We conducted a cross-sectional study of 255 Japanese elderly men aged 60–69 years who participated in annual health check-ups in 2014–2015. The study population was categorized on the basis of a median value of HGF of 300.6 pg/mL.

**Results:**

Among present study population, 128 participants showed low HGF. For participants with low HGF, hemoglobin concentration showed no significant association with handgrip strength (standardized parameter estimate (*β*) = 0.03, *p* = 0.767), but for those with high HGF, hemoglobin concentration was significantly positively associated with handgrip strength (*β* = 0.23, *p* = 0.014).

**Conclusions:**

A significant positive association between hemoglobin level and handgrip strength was established for elderly Japanese men aged 60–69 years with high HGF but not for participants with low HGF. Our finding indicates that HGF levels could determine the relationship of hemoglobin concentration with handgrip strength in elderly Japanese men aged 60–69 years. This result can be expected to serve as an effective tool for the clarification of the roles played by HGF and hemoglobin concentration in maintenance of muscle strength.

## Introduction

Hepatocyte growth factor (HGF), which is secreted from muscle fibers following muscle injury [1], is a factor that is known to be capable of activating satellite cells directly [2, 3]. Therefore, a high concentration of HGF might indicate a higher activity of muscle repair whereas a low concentration of HGF might indicate a lower activity.

On the other hand, a reduction in hemoglobin concentration was previously reported to be directly associated with a decline in handgrip strength (< 26 kg) among elderly Australian men aged ≥ 70 years [4]. Another study with elderly participants aged ≥ 65 years reported that participants with anemia defined by hemoglobin level showed significantly lower handgrip strength than those without anemia [5]. Hemoglobin concentration may thus be directly associated with handgrip strength. However, the background mechanism that governs this association has not yet been clarified.

Since hemoglobin concentration is unlikely to have a direct influence on maintaining muscle strength, unlike HGF which regulates the muscle satellite cells [2, 3], the positive effect of hemoglobin concentration on the prevention of a decline in muscle strength should be supportive.

We therefore hypothesized that the positive association between hemoglobin concentration and handgrip strength would be especially evident for participants with high HGF, since they can be expected to have a higher activity level of muscle repair and the supportive effect of hemoglobin concentration to be more prominent.

Aging is the most frequent cause of a reduction in muscle strength [6] and development of anemia [7]. Therefore, clarification of the association between hemoglobin and handgrip strength in relation to HGF levels among elderly participants can be expected to serve as an effective tool for elucidating the role of the background mechanism that governs age-related muscle strength reduction.

To clarify and validate this hypothesis, we conducted a cross-sectional study of 255 elderly Japanese men aged 60–69 years who participated in general health check-ups in 2014–2015.

## Materials and methods

### Study population

The influence of age-related bone marrow activity decline [7] might act as strong confounding factor for the analyses performed for this study, which aimed to clarify the association between hemoglobin concentration and handgrip strength in relation to HGF levels. Therefore, we conducted a cross-sectional study with participants from a narrow age range, which showed no significant association between age and hemoglobin concentration.

The total number of male residents aged 60–69 of Goto City (estimated by the National Institute of Population and Social Security Research in March 2013) was 3264 in 2015 [8]. The study population comprised 272 male residents aged 60–69 years from the Goto Islands located in the western part of Japan, who underwent an annual medical check-up in 2014 and 2015 as recommended by the Japanese government. Those without data for HGF (*n* = 2) were excluded from the study population. To avoid the effects of chronic inflammatory disease and paralysis caused by stroke, participants with high white blood cell count (WBC) (≥ 10,000 cells/μL) (*n* = 1) and history of stroke (*n* = 14) were also excluded from the analysis. The remaining participants, 255 men with a mean age of 65.4 years (standard deviation (SD) 2.6, range 60–69) were enrolled in the study.

### Data collection and laboratory measurements

Handgrip strength was recorded as the grip strength from two measurements performed with each hand using a handgrip dynamometer (Smedley, Matsumiya Ika Seiki Seisakujo, Tokyo, Japan), with the maximum value used.

Body weight and height were measured with an automatic body composition analyzer (BF-220; Tanita, Tokyo, Japan), after which body mass index (BMI, kg/m^2^) was calculated.

Systolic (SBP) and diastolic (DBP) blood pressures of the right arm were measured with a blood pressure measuring device (HEM-907; Omron, Kyoto, Japan) after the participants had remained in a sitting position for at least 5 min of rest and recorded by a trained examiner.

Fasting blood samples were collected in a EDTA-2K tube, a siliconized tube, and a sodium fluoride tube. Samples from the EDTA-2K tube were used to measure WBC and hemoglobin (Hb) concentration with an automated procedure at SRL, Inc. (Tokyo, Japan). Serum triglycerides (TG), serum high-density lipoprotein cholesterol (HDLc), serum γ-glutamyltranspeptidase (γ-GTP), hemoglobin A1c (HbA_1C_), and serum creatinine (Cre) were also measured with standard laboratory procedures at SRL, Inc. (Tokyo, Japan). The glomerular filtration rate (eGFR) was estimated with an established method recently adapted and introduced by a working group of the Japanese Chronic Kidney Disease Initiative [9], to yield an estimate of eGFR (ml/min/1.73 m^2^) = 194 × (serum Cre (enzyme method))^−1.094^ × (age)^−0.287^.

To measure HGF, serum samples were first diluted fourfold with specific Bio-Plex sample diluents and then analyzed for HGF by using a multiplexed fluorescent magnetic bead-based immunoassay (Bio-Rad Laboratories, Inc., Hercules, CA, USA), according to the manufacturer’s instructions [10]. If the serum values were outside the detection range, they were replaced with the lower or upper detection limits. Error bars were used to indicate SD of the means. *P* values were derived from two-tailed Welch’s t-test.

### Statistical analysis

Characteristics of the study population in relation to HGF levels were expressed as mean ± SD except for TG, γ-GTP, and HGF. Since these three factors showed a skewed distribution, the characteristics of the study population were expressed as median [first quartile and third quartile], followed by logarithmic transformation. The regression model for mean values was used for calculating *p* values.

A simple correlation analysis and multiple linear regression analysis of handgrip strength were conducted with relevant factors adjusted for confounding factors based a median value for HGF levels (300.6 pg/mL) since we did not know the exact cutoff point for the analyses. Previous studies reported that both HGF and hemoglobin concentration are independently associated with vascular remodeling [11–14]. We therefore included cardiovascular risk factors as confounding factors for the analysis used in the current study. Furthermore, alcohol consumption and smoking status are well known as factors that affect vascular remodeling. Since γ-GTP has been recognized as a factor that is influenced by alcohol consumption [15], and WBC as a factor that is influenced by smoking status [16], we added γ-GTP and WBC as confounding factors to our analysis instead of using alcohol consumption and smoking status directly as was done in a previous study of ours [11, 17]. For the multiple linear regression analysis, adjustments were made for age, SBP (mmHg), BMI (kg/m^2^), TG (mg/dL), HDLc (mg/dL), γ-GTP (U/L), HbA1c (%), eGFR (mL/min/1.73 m^2^), and WBC (cells/μL).

We also evaluated the effect of HGF categories (low and high) on the association between hemoglobin and handgrips strength by linear regression analysis.

All statistical analyses were performed with the SAS system for Windows (version 9.4; SAS Inc., Cary, NC). As was done in a previous study [18], values of *p* < 0.05 for main effects and *p* < 0.2 for interactions were considered to be statistically significant.

## Results

### Characteristics of study population based on HGF levels

For the total study population of 255 participants, age showed no significant association with HGF (simple correlation coefficient (*r*) = 0.02, *p* = 0.797) and hemoglobin (*r* = − 0.07, *p* = 0.269) and a significant inverse association with handgrip strength (*r* = − 0.25, *p* < 0.001). High HGF (> 300.6 pg/mL) was observed in 127 participants. They also showed significantly higher values for BMI, TG, γ-GTP, WBC, and hemoglobin concentration and significantly lower values for HDLc compared with participants with low HGF (Table 1).

**Table 1:** Characteristics of the low and high (HGF) level groups of the study population

	Low HGF (≤ 300.6 pg/mL)	High HGF (> 300.6 pg/mL)	*p*
No. of participants	128	127	
Age, years	65.4 ± 2.5	65.4 ± 2.6	0.926
Systolic blood pressure (SBP), mmHg	135 ± 18	137 ± 17	0.299
Diastolic blood pressure (DBP), mmHg	85 ± 12	85 ± 11	0.691
Body mass index (BMI), kg/m^2^	23.1 ± 2.8	24.2 ± 3.0	0.002
Serum triglycerides (TG), mg/dL	84 [66–119]^*^	103 [75–134]^*^	0.012^**^
Serum HDL-cholesterol (HDLc), mg/dL	60 ± 14	55 ± 14	0.007
Serum γ-glutamyltranspeptidase (γ-GTP), U/L	31 [20–46]^*^	36 [24–59]^*^	0.020^**^
Hemoglobin A1c (HbA1c), %	5.6 ± 0.6	5.7 ± 0.7	0.312
Glomerular filtration rate (eGFR), mL/min/1.73m^2^	74.5 ± 13.2	71.7 ± 13.8	0.104
White blood cell (WBC), cells/μL	5466 ± 1492	5981 ± 1349	0.004
Hepatocyte growth factor (HGF), pg/mL	221.0 [177.9–262.9]^*^	422.4 [357.1–536.8]^*^	< 0.001^**^
Handgrip strength, kg	38.8 ± 6.4	38.6 ± 5.6	0.842
Hemoglobin, g/dL	14.5 ± 1.1	14.8 ± 1.1	0.032

Values: mean ± standard deviation

*Median values [first quartile, third quartile]. Regression model for mean values was used for determining *p* values

**Logarithmic transformation was used for evaluating *p*

### Association between HGF concentration and handgrip strength for all participants

**Fig. 1:**
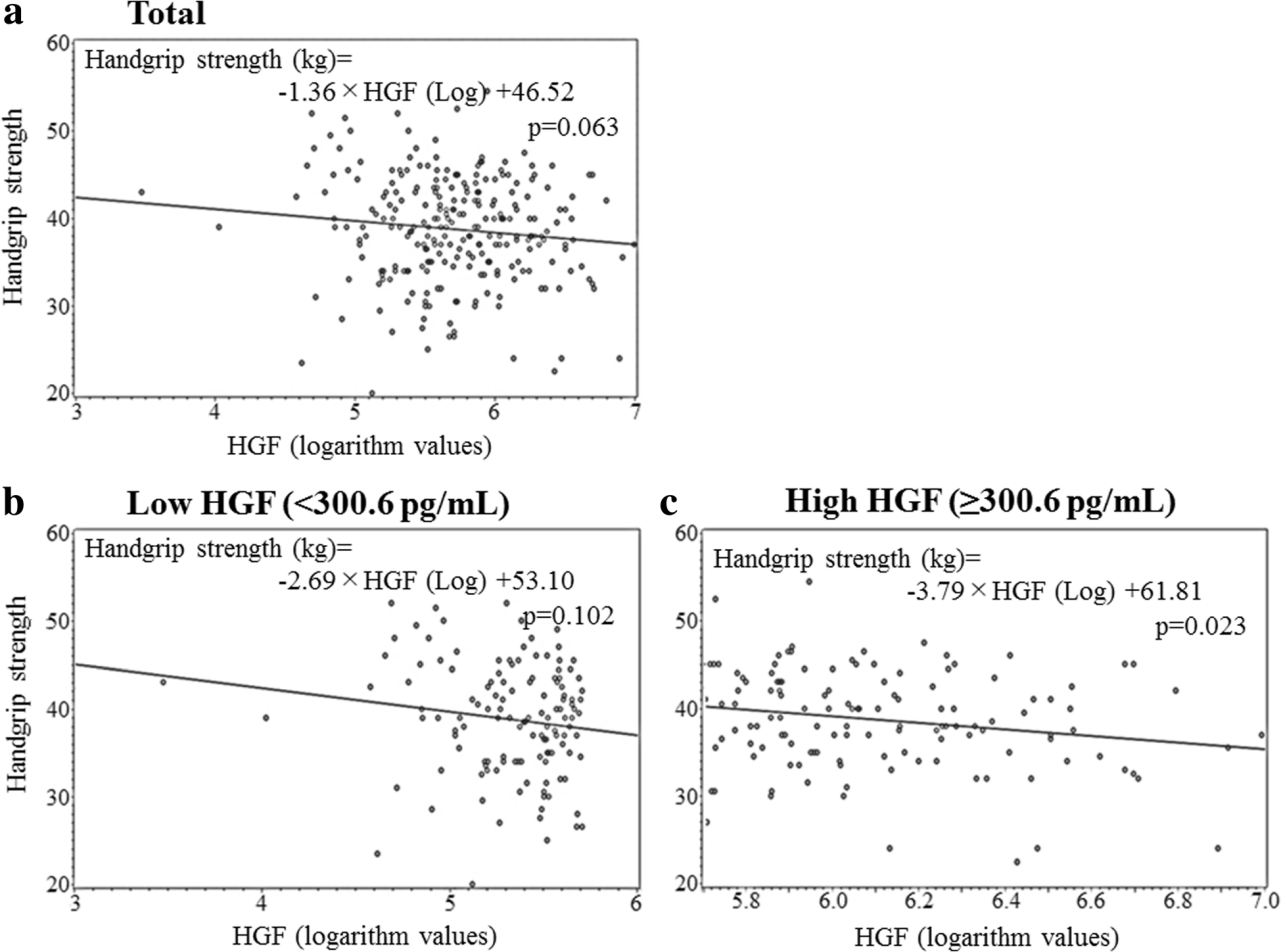
Scatter plot of handgrip strength and hepatocyte growth factor (HGF) for participants with **a** total, **b** low hepatocyte growth factor (HGF), and **c** high HGF

No significant associations between HGF concentration and handgrip strength were observed (Table 2, Fig. 1).

**Table 2:** Simple correlation analysis and multiple linear regression analysis of handgrip strength with relevant factors adjusted for confounding factors

	*r* (*p*)	Hemoglobin (−)			Hemoglobin (+)		
		HGF (+)			HGF (−)		
		*Β*	*β*	*p*	*Β*	*β*	*p*
No. of participants	255						
Age, years	− 0.25 (< 0.001)	− 0.58	− 0.25	< 0.001	− 0.57	− 0.25	< 0.001
Systolic blood pressure (SBP)	0.13 (0.044)	0.05	0.15	0.019	0.05	0.14	0.023
Body mass index (BMI)	0.06 (0.378)	0.22	0.11	0.099	0.14	0.07	0.306
Serum triglycerides (TG)	− 0.03 (0.592)	− 0.07	− 0.01	0.937	− 0.16	− 0.01	0.858
Serum HDL-cholesterol (HDLc)	0.11 (0.085)	0.04	0.10	0.194	0.05	0.11	0.148
Serum γ-glutamyltranspeptidase (γ-GTP)	− 0.07 (0.282)	− 0.87	− 0.10	0.148	− 1.04	− 0.11	0.085
Hemoglobin A1c (HbA1c)	− 0.07 (0.237)	− 0.88	− 0.09	0.147	− 0.80	− 0.08	0.188
Glomerular filtration rate (eGFR)	− 0.15 (0.014)	− 0.06	− 0.14	0.019	− 0.07	− 0.15	0.017
White blood cell (WBC)	0.01 (0.861)	0.00004	0.01	0.876	− 0.0002	− 0.04	0.532
Hemoglobin	0.13 (0.032)	–	–	–	0.68	0.13	0.0497
Hepatocyte growth factor (HGF)	− 0.12 (0.063)	− 1.27	− 0.11	0.085	–	–	–

TG, γ-GTP, and HGF were calculated as logarithm values

*r* (*p*) simple correlation coefficient (*p* factor), *Β* parameter estimate, *β* standardized parameter estimate, *p p* factor for multivariable linear regression models, *Hemoglobin (−) HGF (+)* relevant factors without hemoglobin and with HGF, *Hemoglobin (+) HGF (−)* relevant factors with hemoglobin and without HGF

### Association between hemoglobin concentration and handgrip strength for all participants

**Fig. 2:**
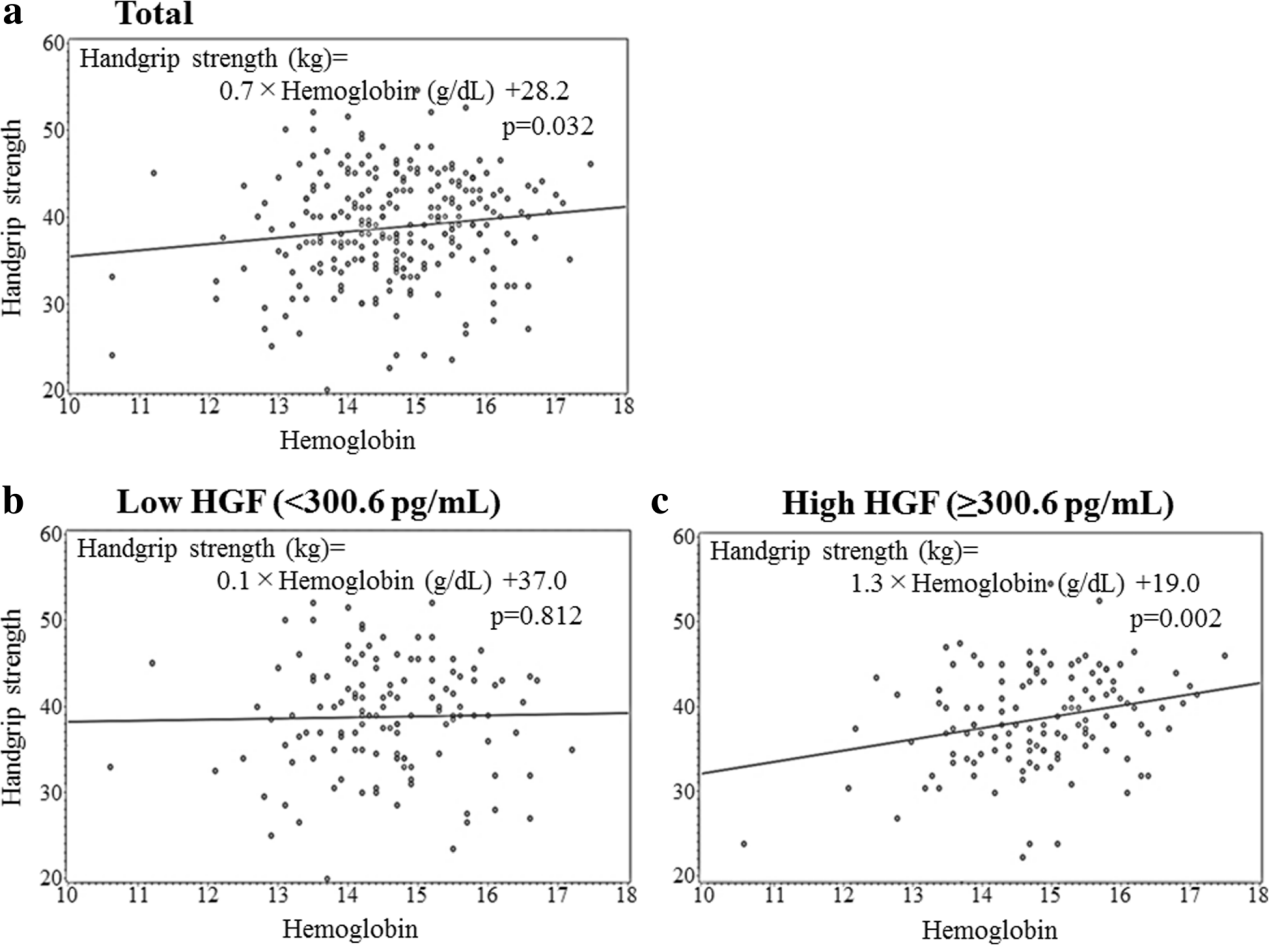
Scatter plot of handgrip strength and hemoglobin for participants with **a** total, **b** low hepatocyte growth factor (HGF), and **c** high HGF

As shown in Table 2 and Fig. 2, hemoglobin concentration was slightly but significantly associated with handgrip strength. This association remained unchanged even after further adjustments for known cardiovascular risk factors.

### Association between HGF concentration and handgrip strength in relation to HGF levels

The results of simple correlation analysis showed that for participants with high HGF, HGF concentration was significantly and inversely associated with handgrip strength (Table 3, Fig. 1) whereas for participants with low HGF, no significant correlation was observed. For participants with high HGF, even though simple correlation analysis showed a significant inverse association between HGF and handgrip strength, after adjustment for other variables, the association became insignificant (Table 3).

**Table 3:** Simple correlation analysis and multiple linear regression analysis of handgrip strength with relevant factors (including HGF) adjusted for confounding factors for high and low HGF levels

	Low HGF (≤ 300.6 pg/mL)				High HGF (> 300.6 pg/mL)			
	*r* (*p*)	*Β*	*β*	*p*	*r* (*p*)	*Β*	*β*	*p*
No. of participants	128				127			
Age, years	− 0.23 (0.008)	− 0.52	− 0.20	0.016	− 0.28 (0.002)	− 0.68	− 0.32	< 0.001
Systolic blood pressure (SBP)	0.14 (0.118)	0.05	0.14	0.115	0.11 (0.203)	0.04	0.13	0.170
Body mass index (BMI)	0.08 (0.398)	0.34	0.15	0.106	0.04 (0.641)	0.07	0.04	0.698
Serum triglycerides (TG)	− 0.13 (0.158)	− 1.27	− 0.09	0.359	0.06 (0.491)	0.81	0.08	0.508
Serum HDL-cholesterol (HDLc)	0.14 (0.106)	0.06	0.14	0.162	0.07 (0.442)	0.01	0.03	0.780
Serum γ-glutamyltranspeptidase (γ-GTP)	− 0.19 (0.031)	− 2.08	− 0.21	0.022	0.07 (0.442)	0.13	0.02	0.873
Hemoglobin A1c (HbA1c)	− 0.007 (0.940)	0.09	0.01	0.929	− 0.14 (0.117)	− 1.53	− 0.18	0.049
Glomerular filtration rate (eGFR)	− 0.22 (0.012)	− 0.09	− 0.19	0.027	− 0.08 (0.353)	− 0.03	− 0.07	0.444
White blood cell (WBC)	0.03 (0.021)	0.0003	0.06	0.499	− 0.006 (0.944)	− 0.0001	− 0.03	0.730
Hepatocyte growth factor (HGF)	− 0.15 (0.102)	− 2.55	− 0.14	0.109	− 0.20 (0.023)	− 3.02	− 0.16	0.078

TG, γ-GTP, and HGF are calculated as logarithm values

*r* (*p*) simple correlation coefficient (*p* factor), *Β* parameter estimate, *β* standardized parameter estimate, *p p* factor for multivariable linear regression models

### Association between hemoglobin concentration and handgrip strength in relation to HGF levels

For participants with high HGF, hemoglobin concentration was significantly positively associated with handgrip strength while for participants with low HGF no significant association was observed (Table 4, Fig. 2). These associations remained unchanged even after adjustments for known cardiovascular risk factors (Table 4).

**Table 4:** Simple correlation analysis and multiple linear regression analysis of handgrip strength with relevant factors (including hemoglobin) adjusted for confounding factors for high and low HGF levels

	Low HGF (≤ 300.6 pg/mL)				High HGF (> 300.6 pg/mL)			
	*r* (*p*)	*Β*	*β*	*p*	*r* (*p*)	*Β*	*β*	*p*
No. of participants	128				127			
Age, years	− 0.23 (0.008)	− 0.55	− 0.22	0.010	− 0.28 (0.002)	− 0.64	− 0.30	0.001
Systolic blood pressure (SBP)	0.14 (0.118)	0.04	0.12	0.149	0.11 (0.203)	0.05	0.14	0.127
Body mass index (BMI)	0.08 (0.398)	0.33	0.14	0.129	0.04 (0.641)	− 0.06	− 0.03	0.749
Serum triglycerides (TG)	− 0.13 (0.158)	− 1.01	− 0.07	0.471	0.06 (0.491)	0.66	0.06	0.583
Serum HDL-cholesterol (HDLc)	0.14 (0.106)	0.07	0.15	0.139	0.07 (0.442)	0.01	0.02	0.844
Serum γ-glutamyltranspeptidase (γ-GTP)	− 0.19 (0.031)	− 2.10	− 0.21	0.023	0.07 (0.442)	0.05	0.01	0.952
Hemoglobin A1c (HbA1c)	− 0.007 (0.940)	0.12	0.01	0.905	− 0.14 (0.117)	− 1.47	− 0.17	0.056
Glomerular filtration rate (eGFR)	− 0.22 (0.012)	− 0.10	− 0.20	0.020	− 0.08 (0.353)	− 0.04	− 0.10	0.272
White blood cell (WBC)	0.03 (0.021)	0.0001	0.03	0.760	− 0.006 (0.944)	− 0.0004	− 0.10	0.282
Hemoglobin	0.02 (0.812)	0.16	0.03	0.767	0.27 (0.002)	1.12	0.23	0.014

TG and γ-GTP are calculated as logarithm values

*r* (*p*) simple correlation coefficient (*p* factor), *Β* parameter estimate, *β* standardized parameter estimate, *p p* factor for multivariable linear regression models

### Effect of associations between hemoglobin level and two HGF categories on handgrip strength

An investigation into the effects of the associations between hemoglobin level and the two HGF categories (high and low) on handgrip strength revealed a significant interaction: *p* values for the effect of this interaction were *p* = 0.074 for the crude model and *p* = 0.142 for the fully adjusted model.

### Association between hemoglobin concentration and handgrip strength in relation to HGF level quartile

To check the statistical effect of changes in the association between hemoglobin and handgrip strength in terms of HGF concentration, we also evaluated those associations in relation to HGF level quartile (Q1:< 221.9 pg/dL (*n* = 64), Q2:221.9–300.6 pg/dL (*n* = 64), Q3: 300.7–418.7 pg/dL (*n* = 63), Q4:≥ 418.8 pg/dL (*n* = 64)). Simple correlation analysis showed that, although significant associations were observed for the highest (Q4) (simple correlation coefficient (*r*) = 0.26, *p* = 0.039) and second highest (Q3) HGF levels (*r* = 0.30, *p* = 0.017), no significant associations were observed for the second lowest (Q2) (*r* = − 0.01, *p* = 0.939) and lowest (Q1) levels (*r* = 0.08, *p* = 0.546) (Table 5).

**Table 5:** Simple correlation analysis of hemoglobin and handgrip strength in relation to quartiles of HGF levels

	Q1 (< 221.9 pg/dL)	Q2 (221.9–300.6 pg/dL)	Q3 (300.7–418.7 pg/dL)	Q4 (≥ 418.8 pg/dL)
	*r* (*p*)	*r* (*p*)	*r* (*p*)	*r* (*p*)
No. of participants	64	64	63	64
Hemoglobin	0.08 (0.546)	− 0.01 (0.939)	0.30 (0.017)	0.26 (0.039)

*r* (*p*) simple correlation coefficient (*p* factor)

## Discussion

The major finding of our study is that, independent of known cardiovascular risk factors, hemoglobin is positively associated with handgrip strength among elderly men aged 60–69 years, especially participants with high HGF.

Oxidative stress is one of the most important causes of the development of age-related diseases [19] including reduction of muscle strength [6]. Since hemoglobin concentration constitutes an important component of the antioxidant capacity of blood [20, 21], anemia as defined by hemoglobin levels could be associated with lower muscle strength among elderly participants [5] since it may indicate a reduction in antioxidant capability. In fact, a previous Australian study of elderly men found that a reduction in hemoglobin concentration was directly associated with a decline in handgrip strength [4].

Overall, the participants enrolled in the present study showed a slight, but nevertheless significant and positive association between hemoglobin concentration and handgrip strength. A further analysis of our findings stratified by HGF levels indicated that this significant and positive association was limited to participants with high HGF concentrations.

The aging process is characterized by an imbalance between an increase in the production of reactive oxygen species in organisms and antioxidant defenses as a whole [4, 5, 19–21]. Furthermore, HGF is involved in the regulation of skeletal muscle satellite cell proliferation and differentiation [2, 3]. Finally, tissue injury, which is associated with production of reactive oxygen species, has been found to activate satellite cells via the HGF regulating pathway [22]. This means that participants with high HGF levels may be capable of a higher production of reactive oxygen species. Therefore, even if handgrip strength measurements show essentially the same values for participants with low (38.8 ± 6.4) and high HGF (38.6 ± 5.6) concentrations (*p* = 0.842) as shown in the present study, the need for antioxidant activity could be much stronger for participants with high HGF than with low HGF concentrations. For the participants with high HGF levels, HGF showed significantly inverse association with handgrip strength, but after further adjustments for other variables, this association was no longer observed. These findings may constitute support for the existence of the abovementioned background mechanism because the differences in associations seem to indicate that higher levels of HGF had a beneficial effect on maintaining muscle strength for participants with higher risk and that such an effect disappeared after adjustment for the various risk factors.

Finally, since hemoglobin has antioxidant capacity [20, 21], the importance of the presence and concentration of hemoglobin should be much greater for participants with high HGF than with low HGF.

Since it was reported that gender differences have an effect on sarcopenia risk factors for a Japanese population [23], a sex-specific analysis would be mandatory for the present analysis. Moreover, age-related muscle injury might stimulate HGF production because HGF regulates satellite cells [2, 3], so that age could influence HGF levels. In addition, since age-related reduction in bone marrow activity lowers hemoglobin levels [7], age could also affect hemoglobin levels. In view of these considerations, we enrolled a target population restricted to men within a narrow age range. Since our present study aimed to clarify the HGF-level-specific association between hemoglobin and age-related muscle strength reduction, the population of a similar age enrolled in our study showed a significantly inverse association with handgrip strength, while no significant associations were observed between HGF levels and hemoglobin. This constitutes a notable strength of our study.

The clinical implication of the results presented here is that, since the aging process is characterized by an imbalance between an increased in the production of reactive oxygen species and that of antioxidant defenses [4, 5, 19–21], this imbalance could constitute an efficient tool for assessing the influence of aging. And the association between HGF levels and hemoglobin concentration might be a suitable candidate for evaluating the influence of aging on diagnosis in daily clinical practice by indicating such an imbalance. Furthermore, our findings can be expected to serve as an effective tool for the clarification of the roles played by HGF and hemoglobin in maintenance of muscle strength. Since age-related decline in bone marrow activity causes a reduction in hemoglobin [7], clarifying those roles in the case of elderly participants should be an efficient tool for elucidating the background mechanism that governs age-related muscle strength reduction.

Potential limitations of this study warrant consideration. First, the activity of oxygen species in the participants with high HGF in our study could be expected to be higher than that in the participants with low HGF. However, no data was available with regards to evaluation of activity of oxygen species. Further investigation using data for oxygen species is therefore necessary. Second, although we identified significant positive associations between hemoglobin concentration and handgrip strength among participants with high HGF but not for participants with low HGF, the exact cutoff point for this classification remains unknown. Further investigations to clarify the exact mechanism that underlies our findings are thus necessary to determine the exact cutoff point for HGF levels. However, when we performed further analyses using quartile HGF values, a significant positive association between hemoglobin levels and handgrip strength was observed even for participants with the highest (Q4) and second highest (Q3) HGF level quartiles, while no significant associations were observed for the second lowest (Q2) and the lowest (Q1) quartiles. Since we had no access to data for women with HGF and handgrip strength, even though gender differences can be expected to affect associations studied in the present analysis, we could not evaluate those associations for women. Moreover, since this study comprised participants who underwent an annual voluntary health check-up, the influence of selection bias is inevitable. Finally, because this was a cross-sectional study, causal relationships could not be established.

## Conclusion

In conclusion, a significant positive association between hemoglobin concentration and handgrip strength was established for community-dwelling elderly Japanese men aged 60–69 years, especially for participants with relatively higher levels of HGF. Our finding indicates that HGF levels could determine the relationship of hemoglobin concentration on handgrip strength in elderly Japanese men. This result can be expected to serve as an effective tool for the clarification of the roles played by HGF and hemoglobin in maintenance of muscle strength among elderly participants.

## Abbreviations

BMI: Body mass index
Cre: Creatinine
DBP: Diastolic blood pressure
eGFR: Glomerular filtration rate
Hb: Hemoglobin
HbA1c: Hemoglobin A1c
HDLc: High-density lipoprotein cholesterol
HGF: Hepatocyte growth factor
SBP: Systolic blood pressure
TG: Triglycerides
WBC: White blood cell
*β*: Standardized parameter estimate
γ-GTP: γ-Glutamyltranspeptidase
